# Polyelectrolytes Formulated with Primary Unconjugated Bile Acid Optimised Pharmacology of Bio-Engineered Implant

**DOI:** 10.3390/pharmaceutics13101713

**Published:** 2021-10-16

**Authors:** Armin Mooranian, Corina Mihaela Ionescu, Susbin Raj Wagle, Bozica Kovacevic, Daniel Walker, Melissa Jones, Jacqueline Chester, Thomas Foster, Edan Johnston, Sanja Kojic, Goran Stojanovic, Momir Mikov, Hani Al-Salami

**Affiliations:** 1 The Biotechnology and Drug Development Research Laboratory, Curtin Medical School & Curtin Health Innovation Research Institute, Curtin University, Perth, WA 6102, Australia; A.Mooranian@curtin.edu.au (A.M.); c.ionescu@postgrad.curtin.edu.au (C.M.I.); susbinraj.wagle@postgrad.curtin.edu.au (S.R.W.); bozica.kovacevic@postgrad.curtin.edu.au (B.K.); danieljcswalker@gmail.com (D.W.); melissa.a.jones@postgrad.curtin.edu.au (M.J.); j.chester@student.curtin.edu.au (J.C.); thomas.p.foster@student.curtin.edu.au (T.F.); edan.johnston@student.curtin.edu.au (E.J.); 2 Hearing Therapeutics, Ear Science Institute Australia, Queen Elizabeth II Medical Centre, Nedlands, WA 6009, Australia; 3 Faculty of Technical Sciences, University of Novi Sad, Trg Dositeja Obradovica 6, 21000 Novi Sad, Serbia; sanjakojic@uns.ac.rs (S.K.); sgoran@uns.ac.rs (G.S.); 4 Department of Pharmacology, Toxicology and Clinical Pharmacology, Faculty of Medicine, University of Novi Sad, Hajduk Veljkova 3, 21101 Novi Sad, Serbia; mikovmomir@gmail.com

**Keywords:** poly-(4styrene)-sulphonate, poly-(allyl)-amine, primary bile acids, chenodeoxycholic acid, pharmacology, transplants, in vivo studies

## Abstract

Introduction. Several studies have shown that different biomaterials and hydrogels comprising various bile acids such as chenodeoxycholic acid (CDCA), as well as excipients such as poly-(styrene)-sulphonate (PSS) and poly-(allyl)-amine (PAA), exhibited positive biological effects on encapsulated viable pancreatic β-cells. Hence, this study aimed to investigate whether incorporating CDCA with PSS and PAA will optimise the functions of encapsulated pancreatic islets post-transplantation in Type 1 diabetes (T1D). Methods. Mice were made T1D, divided into two equal groups, and transplanted with encapsulated islets in PSS-PAA (control) or with CDCA-PSS-PAA (treatment) microcapsules. The effects of transplanted microcapsules on blood glucose, inflammation and the bile acid profile were measured post-transplantation. Results and Conclusion. Compared with control, the treatment group showed better survival rate, improved glycaemic control, and lower inflammatory profile, illustrated by ↓ interleukin 1-β, interleukin-6, interleukin-12, and tumour-necrosis factor-α, and ↓ levels of the bile acid, as well as lithocholic acid in the plasma, liver, large intestine and faeces. The results suggest that CDCA incorporation with PSS-PAA microcapsules exerted beneficial effects on encapsulated islets and resulted in enhanced diabetes treatment, post-transplantation, at the local and systemic levels.

## 1. Introduction 

Harvesting healthy viable islets from human cadavers and transplanting them into Type 1 diabetic patients (T1D) has been considered as an alternative strategy to replace insulin therapy in treating the disease. The transplanted viable islets are meant to release sufficient insulin and enable normalisation of glucose homeostasis and treatment of T1D, based on body’s natural insulin needs. However, wide clinical applications of islet transplantation in patients with T1D have not been accomplished, due to many limitations including host immune response and inflammation, limited sources of healthy islets ready for transplantation, and poor islet survival and functions post-transplantation [1]. One of the ways to improve the survival and functions of transplanted viable islets is via encapsulation with matrices that can provide physical support and protect the islets from the immune system, while allowing for nutrients and gas exchange to take place between the islets and extracellular biological fluids and, hence, supporting islets’ viability and functions post-transplantation. Biomaterials and islet-encapsulating matrices have been widely researched [2,3,4]. Polyelectrolytes, poly-(4styrene)-sulphonate (PSS), poly-allylamine (PAA), as well as some bile acids such as chenodeoxycholic acid (CDCA), have been suggested as potential biomaterials and islet-supporting matrices in islet encapsulation and transplantation, and in T1D treatment. The primary bile acid CDCA possesses unique features including beta-cell positive effects in terms of supporting cell viability and the inflammatory profile, including a reduction in proinflammatory biomarkers. 

Polystyrene sulfonates are a class of medicine that have been used to treat high levels of potassium in blood. They act as potassium chelating agents in disorders including chronic kidney diseases. PSS is considered a polyelectrolyte and has been widely studied as an excipient and as a biologically active compound. In addition, multiple published studies in the literature have demonstrated PSS applications as formulation excipients to form scaffolds and hydrogels suitable for the delivery of drugs and/or biological molecules. Vongsetskul et al. studied the interaction of cationic surfactants and PSS at the DNA level, and correlated such interaction with air/water interface formation of matrices based on the chemical bond formation and molecular arrangement of excipients as a result of surface charges, molecular size and particle arrangement within the formed matrices. The authors concluded that in the presence of PSS, surfactants are rearranged within the matrix; hence, PSS presence can exert significant structural effects on the matrix, forming a multi-layered structured system and significantly influencing the physicochemical and relevant properties of the final formed formulation [5]. With regard to formulation excipients or biological effects at the cellular levels, PSS-based compounds have often been researched in conjunction with PAA. V Moby et al. explored multi-layered 3D structures of matrices comprising PSS/PAA-like building blocks, as a way to support cellular growth and proliferation on transplants. The authors concluded that such 3D structures significantly improved cellular viability, proliferation and functions due to solid and well-defined matrix formation supporting the monolayer development of supporting cells on the transplants, indicating the beneficial effects of such structures in cell growth and transplantation outcomes [6]. In addition to exploring biological effects of PSS and PAA, studies have also explored the application of PAA alone. 

Polyallylamine-based compounds and chemical moieties are cationic polyelectrolytes produced by allylamine polymerisation. They have been used to form various 3D structures for biomedical and cellular applications, alone or combined with other polyelectrolytes. In a review by Rokstad et al., the authors explored various aspects of cell encapsulation in clinical settings. Functionalisation of biomaterials was considered as one of the effective pathways to cell encapsulation and functions, long-term, and the applications of polyelectrolytes were considered to be fundamental to graft optimisation processes [7]. In a recent study by de Sa et al., the authors explored the applications of PAA in producing nanostructured proteins as layer-by-layer thin films for medical and implantable applications, and suggested wide applications of PAA combined with other materials, such as bovine serum albumin proteins, in nano fabrication and matrix formation [8]. Moreover, P Andreozzi et al. explored the applications of PAA phosphate in the nanocarrier delivery of RNA. As a proof-of-concept, the authors investigated the applications of PAA phosphate in silencing RNA bio-nanotechnologies, as a way of utilising nanodelivery systems for intracellular targeting, and also considered ways to stabilise the nanocarriers at physiological pH conditions and to enable optimum nanocarrier release kinetics. The authors concluded that PAA phosphate nanocarriers have significant potential applications for the intracellular delivery of genetic materials [9]. In a recent study in our laboratory, when PSS and PAA were added to a biomaterial matrix containing the bile acid CDCA, the matrix provided significant, optimised and desirable biological effects on encapsulated pancreatic β-cells in vitro [10]. Alteration of inflammatory interleukins was observed by encapsulated pancreatic β-cells and such alteration was associated with cell viability, functions and the inflammatory profile.

Accordingly, this study aimed to explore the applications of PSS (1%) and PAA (1%) with or without the bile acid CDCA (1%), in vivo, in the encapsulation, delivery and transplantation of primary and viable islets of Langerhans, in the context of T1D treatment.

## 2. Materials and Methods

For the processes of islet harvesting and formulation preparation, consumables were purchased from Sigma Aldrich (Castle Hill, NSW, Australia), Scharlab S.L (Barcelona, Spain) and ThermoFisher (Scoresby, VIC, Australia) and prepared and stored when not in use, as per our established protocols [11,12,13,14,15,16]. For islet digestion, centrifugation, identification, separation and processing, collagenase digestive enzyme was used via injection into the bile duct and the pancreas of freshly culled mice, as per our approved protocols. For islet harvesting and processing prior to microencapsulation, islets were cultured in freshly prepared and aliquoted RPMI media, and supplemented with 5.5 mM glucose and 10% foetal bovine serum, as well as freshly prepared 1% penicillin (Sigma-Aldrich, Castle Hill, NSW, Australia). Sodium alginate was purchased as low viscosity suitable for cell culture, and was prepared as 1.2%, poly-l-ornithine was prepared as 1%, and ultrasoluble gel was prepared as 4%, PAA was prepared as 2.5% and PSS was prepared as 1%, while CDCA was prepared as 1%. The bile acids lithocholic acid (LCA) and ursodeoxycholic acid (UDCA) were purchased from Sigma Aldrich (Australia). The main vehicle was water and mixtures were heated until 40 °C during preparation. The liquid mixtures were stirred for 7–8 h at body temperature (37 °C) before use. The bathing gel, barium chloride, was used as a solution when using our well-established cell and tissue encapsulation technology, known as the Ionic Gelation Vibration Jet Flow [17,18,19,20], which was developed in collaboration with the pharmaceutical drug company BÜCHI Labortechnik (Flawil, Switzerland). All materials and surgical tools used for islet extraction from balb/c mice were acquired from Able SCIENTIFIC (Perth, WA, Australia). Mice were acquired from the Animal Resources Centre (Murdoch, Bentley, WA, Australia), and all animals were used in accordance with the Australian code of ethics for the use of animals for scientific research. All procedures were approved by the ARC Animal Ethics Committee as well as by Curtin Animal Ethics Committee. For skin suturing and closure post-surgery, sutures from Vilet and Ethicon or Reflex7 skin closure system clips were used as per our animal ethics approved protocols. For measuring biological effects post-transplantation, tissues, faeces and blood were collected into Eppendorf tubes supplied through South Pacific Australia. Blood was collected and plasma analysed as per our well-established methods [18,20,21,22,23,24,25]. For insulin and glucose measurements, kits and glucometers were acquired from Accu-Chek (Roche, Sydney, NSW, Australia), and for insulin ultra-sensitive kits, they were acquired from Mercodia (Uppsala, Sweeden) and used, as per manufacturing instructions, on pooled samples. For the microcapsule imaging analyses, platinum coating was used for the scanning electron microscopy and the energy dispersive X-ray spectrometry, based on established protocols, in the John De Laeter Research Centre (Curtin University, Bentley, Perth, WA, Australia) [26,27,28,29]. The Micro-CT imaging analyses were carried out in the CISRO centre (Bentley, Perth, WA, Australia) as per their established protocols [30,31,32,33,34]. Kits and consumables used for the flow cytometric analyses of inflammatory biomarkers were purchased from BD Bioscience CBA (Franklin Lakes, NJ, USA) and used as per the manufacturer’s instructions. Consumables, materials and organic solvents used for bile acids’ analyses and LCMS measurements in tissues, faeces and plasma were acquired from Merck (Castle Hill, NSW, Australia) and local suppliers as per our well-established processes [11,35,36,37,38,39].

### 2.1. Study Design

**Figure 1:**
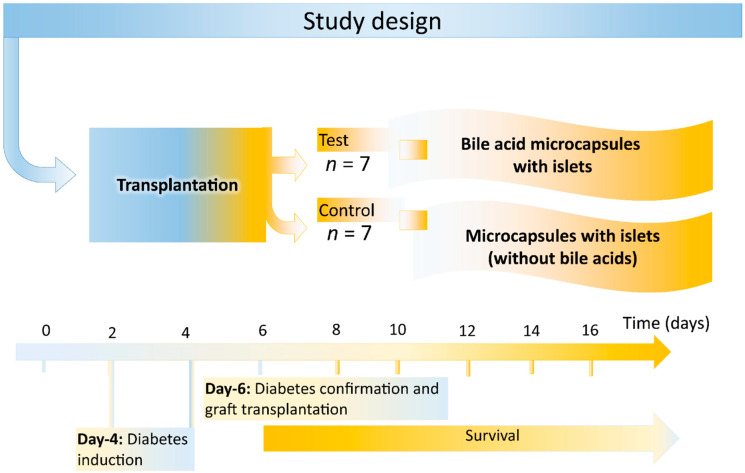
Study design for the transplantation of test (CDCA-PSS-PAA microcapsules with islets) and control (PSS-PAA with islets) in alloxan-induced T1D mice.

The study was designed in order to answer the hypothesis that CDCA-PSS-PAA-based islet microcapsules exerted better antidiabetic and biological effects, compared with PSS-PAA-based islet microcapsules, post-transplantation (Figure 1). Male balb/c mice were injected with alloxan to induce T1D and, once diabetes was confirmed, mice were divided into two equal groups (n = 7, in each group), one was control and transplanted PSS-PAA islets, while the other was treatment and transplanted CDCA-PSS-PAA islets. Each mouse was transplanted ~80 islets, from balb/c donor mice. All procedures were carried out according to the approved guidelines and procedures at Curtin University and the Animal Resources Centre (Australia). Diabetes induction, confirmation and transplantation were carried out by Day 4 and the experiment lasted for 16 days in total. No insulin was used. Upon diabetes confirmation, all mice exhibited severe and significant diabetes symptoms, and hence, the duration of the study was short.

### 2.2. Islet Extraction, Formulation Preparation, and Diabetes Development

Donor mice were euthanized, their internal abdominal cavities were exposed via abdominopelvic incision, and their livers were lifted to uncover the bile duct and the pancreas. The pancreatic tissues were digested, harvested, spread and viable islets were immediately collected under a light microscope using lab pipettes. Upon collection, islets were placed in a rich media in an incubator for 3 h prior to encapsulation and subsequent transplantation into recipient mice. Islets were encapsulated based on our well-established microencapsulation methodologies [39], which were optimised further in this study in order to accommodate for CDCA, PSS and PAA incorporation based on formulation rheological and relevant flow and islet-entrapment properties. The conditions of the encapsulating gelation bath were maintained at a pH of 7.4 and a temperature of 37.5 °C throughout the islet encapsulation and microcapsule formation processes. The concentric nozzle was used, and islets were encapsulated under a nozzle vibration magnitude of 1550 Hz, with the electrode tension set at 685 V. The structure, islet encapsulation efficacy and islet survival were consistent with our previously published studies [20,35,40].

**Figure 2:**
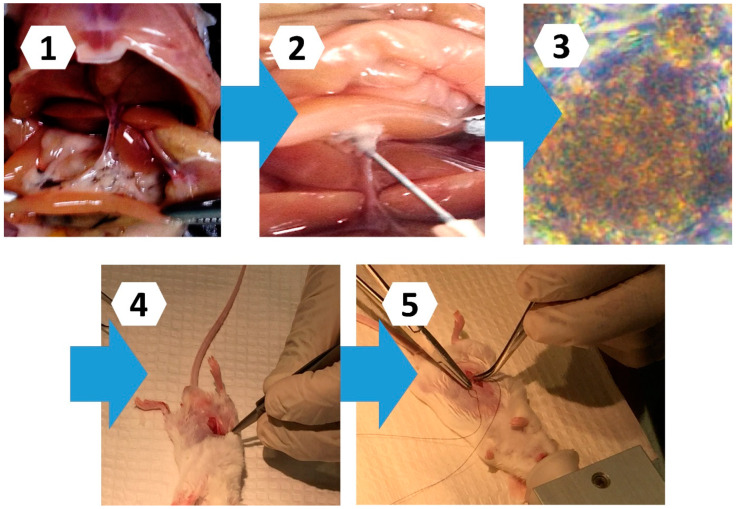
Islet harvesting via (**1**) injection of digestive enzymes through the common bile duct (**2**–**3**), and transplantation of microencapsulated islets into an omentum pouch within the abdominopelvic cavity (**4**), followed by surgical closure of the transplantation site using sutures (**5**) (an illustration surgical procedure).

In both groups of mice, control and treatment, diabetes was induced using our well-established methods [14]. In brief, a single dose of alloxan (150 mg per Kg body weight) was injected subcutaneously or intraperitoneally to recipient mice. The animals became diabetic within two to three days post-alloxan and were provided free access to water and food. Animals were considered T1D if their blood glucose levels were >16 millimolar, had no insulin detected in their blood, and showed common signs and symptoms of the disease. Once T1D was confirmed, mice were transplanted encapsulated islets without CDCA for the control group, or with CDCA for the treatment group. The transplantation surgery was carried out as per our approved protocols by Curtin University Animal Ethics Committee. In brief, upon islet encapsulation and microcapsule formation, diabetic mice were anaesthetized, and their pelvic white fat (visceral fat pads) was identified, isolated, and transplanted with the microcapsules. Upon microcapsules’ fat inoculation, the transplanted region was placed back into the pelvic cavity aseptically. A mixture of antibiotics was applied externally and within the inoculated fat tissues, in order to ensure the prevention of potential internal infection. The peritoneal cavity and the skin were sutured, and additional antibiotics were applied to ensure the prevention of external skin infection. Once the transplantation process was completed according to approved surgical procedures, mice were closely observed to ensure the absence of any surgical complications such as tissue damage, bleeding, and surgical injuries, and that all areas were covered with antibiotics. Once the surgery was completed, each mouse was placed in a specialised incubator designed to ensure the mice were warm, comfortable and in the best recovery conditions to expedite healing and maximise transplantation success. Post-surgery, mice were provided with easy access to soft food and water. The surgical transplantation was conducted according to our approved ethics application. Monitoring sheets were used to ensure best surgical outcome and transplant performance post-surgery, based on our approved ethics (Figure 2).

### 2.3. Topographic and Surface Elemental Composition, Size Distribution, Water Uptake, and Integrity and Intactness Analyses

Both types of microcapsules, control and treatment, were analysed for topography, surface elemental composition, size distribution, water uptake, and integrity and intactness profiles under stress. 

For the topographic and surface elemental composition, the instrumentations used were Micro-CT, scanning electron microscopy, light imaging microscopy, confocal imaging and energy dispersive X-ray spectroscopy. For Micro-CT analysis, the Micro-CT 11.5 µm pixel (Bruker Optics, Billerica, MA, USA) system was used, while for the scanning electron microscopy, the Zeiss Neon 40EsB (Zeiss, Oberkochen, Germany) was used. For the light imaging, the Olympus IX-51 LM (Olympus, Tokyo, Japan) was used, while for confocal microscopy, the Nikon A1 confocal system (Nikon, Tokyo, Japan) was used. For energy dispersive X-ray spectroscopy, the Oxford Instruments Aztec X-Act (Oxford, Abingdon, UK) was used. Samples analyses were carried out using our established methods [21,35]. For size distribution, the Mastersizer 2000 (Malvern, UK) was used, while for water uptake assessment, integrity and intactness assessments, tracking of weight change 7 days post-drying, and the count of microcapsules that broke (as a percentage of the total number analysed) after constant shear stress was applied, the Multishaker PSU 20 (Bueco, Hamburg,, Germany) was used. The used methods were based on our well-described and published studies and microcapsules were analysed within 48 h of formation as per our experimental protocols [12,16,20,30,41].

### 2.4. Blood Glucose and Interleukins Analyses

In order to ascertain the glycaemic control as well as the inflammatory profile of control and CDCA-microencapsulated islets, blood glucose concentrations were measured daily and proinflammatory interleukin plasma concentrations were measured at the end of the experiment, in both groups of mice, control and treatment. Blood glucose measurements were carried out using the tail vein of mice, as per our approved ethics application at Curtin University. For blood glucose concentrations, the Accu-Chek glucose meter was used while for the interleukins measurements, six different inflammatory interleukins were measured in plasma using a specialised kit for interleukin. At the end of the experiment, blood samples were collected, and plasma samples were pooled and processed, using our established protocols via Flow Cytometric Bead Array technology. The Attune Flow Cytometer (Life Technologies, Carlsbad, CA, USA) was deployed for plasma analyses as per our well-established methods [20,42,43,44]. The analysed interleukins were interleukin 1-beta (IL-1β), interleukin-17 (IL-17), interleukin-6 (IL-6), interferon-gamma (IFN-γ), interleukin-12, and tumour necrosis factor-alpha (TNF-α), and samples were analysed from both groups of mice, control and treatment, as per our protocols [45].

### 2.5. Bile Acid Mass Spectroscopic Analyses

For tissue and faecal analyses of the three main bile acids, CDCA, LCA and UDCA, liquid chromatography mass spectrometry (LCMS) was used. The analysed samples of CDCA, LCA and UDCA, were from plasma, brain, small intestine, liver, large intestine, and faeces. Samples were processed and pooled for bile acids’ analyses using our well-described methodologies [20]. Prior to LCMS-sample analysis, samples were diluted with mobile phase and acetonitrile (1:1 ratios). For the LCMS system setup and configuration, the LCMS 2020 Schimadzu system (Schimadzu, Kyoto, Japan) was used and it included a DGU-20A3 prominence attached degasser with a built in SIL-20AC HT Prominence Schimadzu autosampler for automated analysis of samples and data generation. In order to obtain accurate estimated ranges for quality control and standard samples, test runs were carried out for 3 randomly selected different samples from each tissue, blood and faeces set of samples, which ascertained our estimated range of concentrations to be measured. Quality control and standard samples were prepared at concentrations ranging from 1 ng to 500 ng, including three samples within the expected concentration of the bile acid and at least one quality concentration lower than the measured sample, in order to ensure accurate measurements of analysed samples. For sample analysis, the mobile phase was made of methanol and water at concentrations of 65% and 35%, respectively. The pH was adjusted to 3 using either an acid or a base, as appropriate, based on our well-established methods for bile acids analyses [36,38]. Reagents and solvents used for sample preparation, processing and analysis were stored in the refrigerator or at room temperature when not in use. If unused, the remaining solutions and prepared reagents were discarded within 2 weeks of preparation. The samples analyses were carried out using the LCMS system equipped with a C18 column (5 µm particles, 100 mm in length and 2 mm internal diameter) that was purchased from Phenomenex (Torrance, CA, USA). A column guard was used, and this was also purchased from Phenomenex (USA). Our established analytical methods for sample analysis included a flow rate of 0.25 mL per minute of mobile phase with the run time being 15 min per sample, based on our published methods [14].

### 2.6. Statistical Analysis

Statistical data were analysed using a parametric/non-parametric *t* test, or a one-way ANOVA and post hoc Tukey, as appropriate. GraphPad Prism (San Diego, CA, USA) 9.0.2 version was used (USA) and the significance level *p*-value was set as less than 5%.

## 3. Results and Discussion

**Figure 3:**
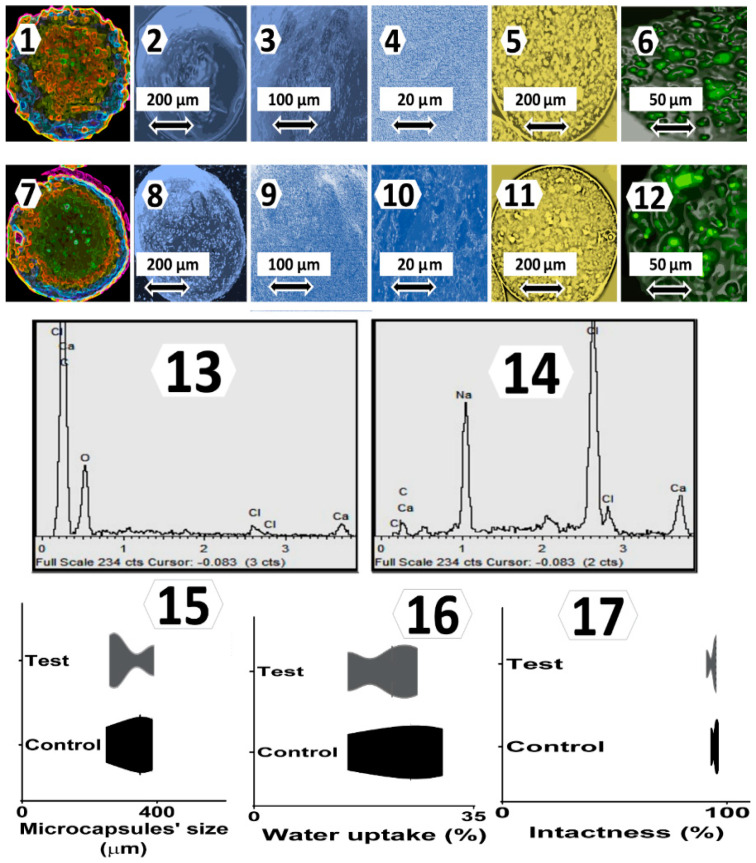
Micro-CT analysis for control (**1**) macrocapsules with SEM micrographs (**2**, **5**, **6**), optical microscopy imaging (**3**) and confocal microscopy (**4**), and micro-CT analysis for treatment (**7**) microcapsules with SEM micrographs (**8**, **11**, **12**), optical microscopy imaging (**9**), and confocal microscopy (**10**). Microcapsules elemental composition of control (**13**) and treatment (**14**), and size distribution (**15**), water uptake (**16**) and intactness under shear stress studies (**17**) are also presented as mean ± SEM.

**Figure 4:**
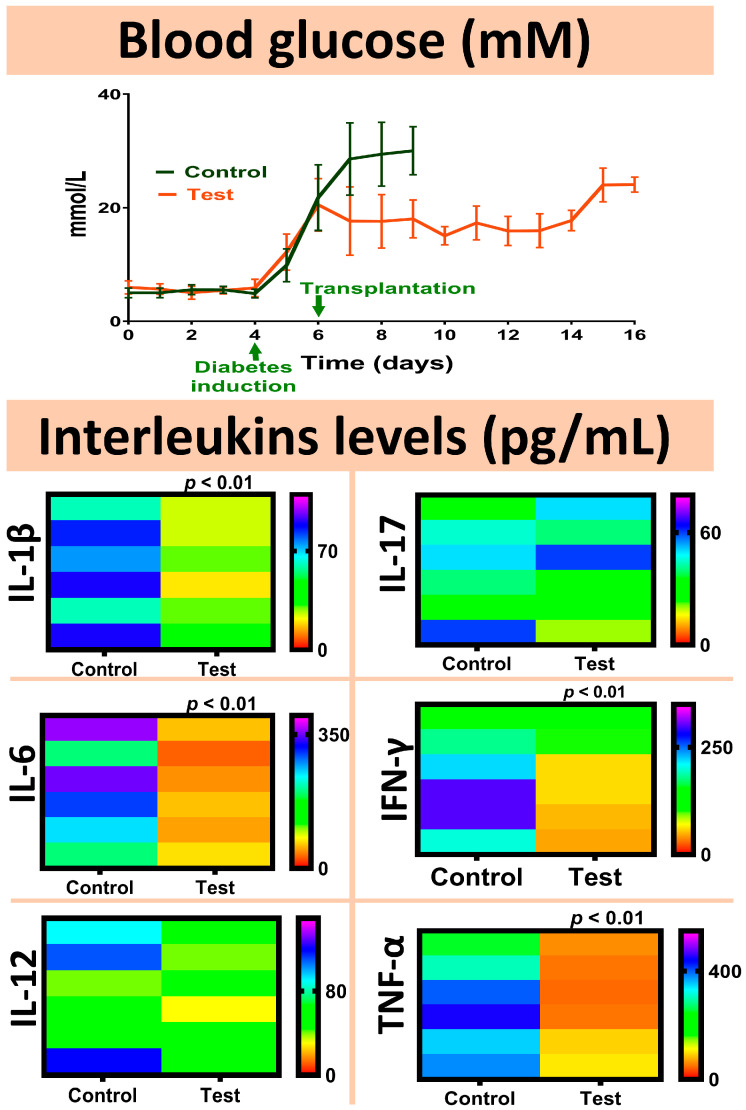
Daily blood glucose concentrations as well as plasma concentrations of IL-1β, IL-6, IL-12, IL-17, IFN-γ and TNF-α. Data are mean ± SEM. (n = 7).

**Figure 5:**
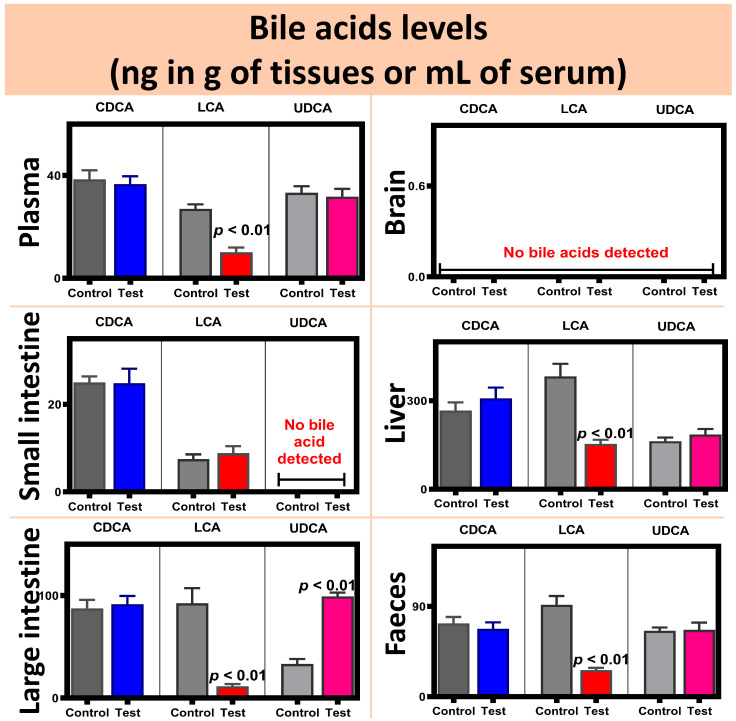
Endogenous bile acid profiling and quantification for CDCA (blue), LCA (red), UDCA (magenta) in plasma, brain, small intestine, liver, large intestine, and faeces of test and control (gray) mice groups. Data are mean ± SEM (n = 7).

**Figure 6:**
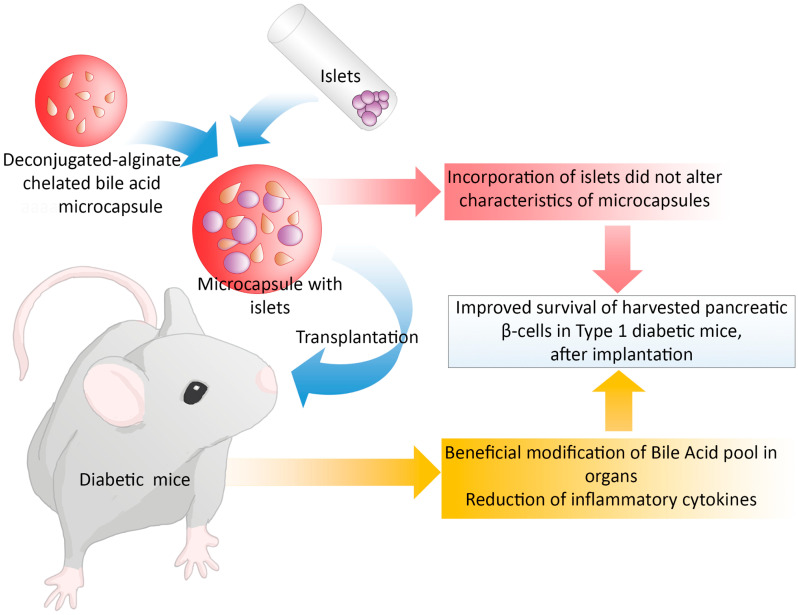
Summery figure of the study design; transplanted experimental microcapsules formed from incorporating islet with polymers and bile acids reduced inflammatory cytokines in vivo.

Topographic and surface elemental composition analyses, microcapsules’ size distribution, microcapsules’ water uptake and resistance, and microcapsules’ integrity and intactness under shear stress assessments are presented in Figure 3. Daily blood glucose measurements as well as interleukins’ concentrations analyses are presented in Figure 4. Bile acids’ concentrations in the plasma, brain, small intestine, liver, large intestine, and faeces samples are presented in Figure 5, while the main findings of the study are summarised and presented in Figure 6.

### 3.1. Topography, Surface Elemental Analysis, Size Distribution, Water Uptake, and Integrity and Intactness

Topographic, morphological and surface analyses by Micro-CT imaging, scanning electron microscopy, surface elemental composition, optical imaging, and microcapsules’ size distribution, of treatment microcapsules vs. control, showed no significant variation in shape, size or surface elemental constituents, which suggests that our microencapsulation method is robust and maintained consistent microcapsule production and islet encapsulation, regardless of CDCA incorporation. The elemental composition was similar, with higher intensities of some atoms compared with others; however, the intensities remained similar overall. Cell distribution within the microcapsules, illustrated by confocal imaging, showed that CDCA-based microcapsules exhibited visible cell content; however, there was no clear or visible difference, compared with control, which suggests that the encapsulation method resulted in similar cellular content in each microcapsule regardless of bile acid addition, and this further supports the robustness and consistency of the method. In addition, the robustness of the deployed microencapsulation method was consistent with our previous studies, demonstrating efficient encapsulation and microcapsules’ contents regardless of bile acid incorporation [26,27]. Microcapsules’ water uptake and their intactness under stress showed no significant variation with or without CDCA incorporation. Lack of CDCA effects on water uptake of microcapsules suggest that CDCA did not exert significant effects on water wettability, evaporation and loss, and did not result in an alteration of microcapsules’ weights over time. The lack of CDCA effects on microcapsules’ intactness is consistent with its observed effects on topography, morphology, elemental composition and water uptake and suggests that CDCA incorporation did not influence the physical or mechanical features of the formed microcapsules. Such effects were anticipated to impact the influence of CDCA on the islet biology and profile within the microcapsules. The anticipated cellular and biological effects were consistent with the literature. Yu et al. investigated cellular uptake and interaction with microcapsules’ excipients, including PAA- and PSS-based ingredients. The authors found that microcapsules’ water uptake and intactness are important features governing the biological performance of microcapsules, as well as their cell biocompatibility and overall functions [46]. Accordingly, at the post-transplantation stage, the biological effects of the microcapsules, including glycaemic control and inflammatory profiles, were both investigated (Figure 4).

### 3.2. Blood Glucose and Interleukins Measurements

In order to assess the biological effects of transplanted microcapsules, concentrations of blood glucose and proinflammatory interleukins and cytokines in plasma were measured (Figure 4). It might be worth mentioning that no insulin was administered to the diabetic mice during the experiment. Daily blood glucose was significantly reduced in the treated group; however, due to the severity of our T1D model and strict adherence to approved animal welfare protocols, the mice did not survive more than a few days post-diabetes induction. The CDCA microcapsules exerted better glycaemic control compared with control, resulting in a longer survival rate of the CDCA-treated mice. The improved survival of the CDCA treated mice suggests that when encapsulated with viable islets, CDCA exerted positive effects on islets’ survival and functions, and subsequently improved insulin secretion and the animal survival rate. However, the diabetes-associated hyperglycaemia was not completely normalised by CDCA-islet transplantation and, hence, diabetes symptoms and hyperglycaemia persisted despite treatment. Consistent with the glycaemic control in the treatment group, the mice showed improved inflammatory profiles and reduced proinflammatory interleukins and cytokines in plasma. Overall, concentrations of the interleukins IL-1β and IL-6, as well as IFN-γ and TNF-α, were all significantly reduced, which suggests that CDCA improved the islets’ survival, and this was at least partly due to its anti-inflammatory effects resulting in improved insulin secretion and reduced overall inflammation. The anti-inflammatory effects of CDCA were potentially due to its direct biological effects on the encapsulated islets or possible systemic effects due its presence in the transplanted microcapsules, although it is likely that the observed anti-inflammatory effect of CDCA was due to its localised effects on the encapsulated islets. It is worth stating that not all measured proinflammatory interleukins were reduced. For example, IL-17 did not significantly change, which suggests that either the anti-inflammatory effect of CDCA was not sufficiently powerful to reduce all the measured interleukins, or perhaps, due the short duration of the study, the CDCA islets did not have sufficient time to reduce all measured interleukins in plasma. Another possibility for the lack of a significant reduction in all proinflammatory interleukins by CDCA treatment is the fact that the cellular secretory pathways of IL-17 as well as IL-12 were not directly impacted on and reduced by CDCA incorporation. This suggests that the hypoglycaemic and anti-inflammatory effects of CDCA were not directly and significantly related to secretory mechanisms of both interleukins, IL-17 and IL-12. Such alterations in levels of interleukins may vary in untreated animals. Published studies have explored the association of IL-12 and IL-17 with other well-known proinflammatory interleukins such as IFN-γ, and their relation with inflammatory diseases. K Tozawa et al. examined the association between IL-12 and IFN-γ in the context of the pathogenesis of colitis development and progression. The authors explored cellular stimulatory and excretory functions of mainly helper T cells of the immune system, in relation with the development and progression of chronic disease. The authors focused on IL-12 and IFN-γ due to their prominence in gut-related inflammatory diseases. The authors concluded that IL-12 induction pathways are different to that of IFN-γ, and that both do not share exact cellular stimulatory processes [47]. In another study by M Pirowska et al., the authors explored concentrations of proinflammatory interleukins including TNF-α, IL-23, and IL-17 in chronic inflammatory and metabolic diseases, with a focus on psoriasis with comorbidities including diabetes and lipid disorders. The authors found that the concentrations of inflammatory interleukins and cytokines are related to the specific condition and stage of the disease, and in patients with diabetes, IL-17 was found to be significantly elevated. The authors concluded that in chronic inflammatory diseases, not all proinflammatory interleukins and cytokines are affected to the same extent, and they depend on wide range of factors including disease severity and glycaemic and lipid haemostatic control, as well as concomitant disorders [48]. This is consistent with previous studies in our laboratory that demonstrated significant alteration in the inflammatory profile due to bile acid-based therapies [20], and that the bile acid profile is closely associated with diabetes induction and development [14]. Accordingly, the bile acid profile was analysed in the plasma, brain, small intestine, liver, large intestine and faeces, and is presented in Figure 5.

### 3.3. Bile Acid Mass Spectroscopic Findings

Bile acid analyses of CDCA, LCA and UDCA showed reduced LCA, in the treatment group, in the plasma, liver, large intestine and faeces compared with control, while no bile acids were detected in the brain and no UDCA was detected in the small intestine in both groups, control and treatment (Figure 5). When comparing the treatment group to the control, the reduction in LCA in plasma suggests a reduction in bile acid metabolism in the liver or gut that results in reduced LCA production (via cholic or chenodeoxycholic acid conjugation pathways [49], which leads to reduced systemically circulating LCA. The reduction in LCA in the liver and large intestine is consistent with plasma and suggests that the reduced catabolism of primary bile acids in the liver and deconjugation in the lower gut contributes to the reduction in LCA in the plasma. In the treatment group, the reduction in LCA concentrations may have been brought about by improved glycaemic and inflammation profiles. This is consistent with the literature. Published studies have shown that LCA concentrations increased in plasma and other tissues, and also in faeces, as a result of T1D development and progression, which suggests significant alteration in the bile acid profile, brought about by diabetes-associated hyperglycaemia and inflammation [14]. Hence, the reduction in LCA concentrations observed in the CDCA-treated group suggests a direct association between improved diabetic symptoms and reduced LCA concentrations, although the reduction did not occur in all analysed tissues, indicating treatment-dependent effects. On the other hand, reduced inflammation and better glycaemic control in the treatment group is consistent with increased UDCA levels in the large intestine, as UDCA supplementation is associated with improved inflammatory profile in diabetes [20]. However, the exact mechanisms and related processes underpinning the direct link between bile acid metabolism, bile acids’ concentrations in tissues, blood and faeces, and concentrations of inflammatory cytokines and interleukins, and the severity of diabetes, remains obscure. It is worth stating that some studies have explored molecular pathways associated with the development of diabetes, the modulation of particular types of proteins, and cellular responses including inflammation. Engin et al. explored the effects of bile acids on the restoration of the unfolded protein response (UPR) in pancreatic β-cells and T1D treatment. The authors found that defects in the expression of mediators of UPR in β-cells can be improved by bile acid administration. The authors concluded that the actions of bile acid were protein-specific and may provide potential therapeutics for prevention or treatment in T1D [50]. Chen et al. investigated the association between UPR and nuclear factor-κB signalling pathways, in the context of inflammation, viability and the functions of β-cells. The authors found that in β-cells, inhibition of stress on the endoplasmic reticulum directly impacted cell viability in a factor-κB-dependent manner. The authors concluded that the cross-talk between the UPR and NFκB signalling pathways is likely to play an important role in inflammation and β-cell death [51]. Accordingly, there are sufficient data in the literature to support at least a sizable correlation between the type of bile acid, the extent of inflammation, and symptoms of diabetes.

## 4. Conclusions

In conclusion, the study aimed at carrying out a preclinical investigation on the potential role of primary bile acids in the delivery of islets when encapsulated in a PAA-PSS microcapsule. The study investigated the formulation characterisation and stability, islet encapsulation efficacy, and islet survival and functions, post-transplantation, using a mouse model of T1D. In addition to assessing the antidiabetic effects of the CDCA-PAA-PSS islet transplant, the study also investigated the impact of the transplant on inflammation and the bile acid profile post-transplantation. The findings showed that CDCA-PAA-PSS transplant exerted optimised glycaemic control and reduced inflammation potentially via bile acid-modulatory effects, demonstrating potential applications of PAA, PSS and CDCA in islet transplantations and diabetes treatment.

## Data Availability

The data presented in this study are available on request from the corresponding author. The data are not publicly available due to author property agreements.
